# Alteration of Cadherin 3 Expression and DNA Methylation in Association with Aggressive Renal Cell Carcinoma

**DOI:** 10.3390/ijms242216476

**Published:** 2023-11-18

**Authors:** Pouriya Faraj Tabrizi, Inga Peters, Inga Schimansky, Natalia Dubrowinskaja, Christel Reese, Hossein Tezval, Markus Antonius Kuczyk, Jürgen Serth

**Affiliations:** 1 Department of Urology and Urological Oncology, Hannover Medical School, 30625 Hannover, Germany; 2 Department of Urology, Krankenhaus Nordwest, 60488 Frankfurt, Germany

**Keywords:** renal cell carcinoma, DNA methylation, cancer epigenetics, *CDH3* expression, DNA methylation

## Abstract

Cadherins (calcium-dependent adhesion proteins) are important in cellular adhesion and may play a role in the development and progression of renal cell carcinoma (RCC). This study investigated changes in cadherin 3 (*CDH3*; P-cadherin) mRNA expression, DNA methylation, and protein expression in RCC and compared the results with the histopathological and clinical characteristics of patients. The possible contribution of CDH3 to tumor cell invasiveness was tested in a functional assay using siRNA-based suppression of *CDH3* expression and subsequent real-time impedance analysis using a Matrigel invasion model. Our analyses revealed a tumor-specific loss of *CDH3* mRNA expression, *CDH3* DNA hypermethylation, and loss of distal tubular and collecting duct CDH3 protein expression in RCC. A relatively higher methylation level in tumors was associated with a loss of cell differentiation and higher clinical stage. siRNA-induced suppression of *CDH3* expression modulated the invasion characteristics of tumor cells in the impedance-based real-time cellular analysis. Our results indicate that loss of *CDH3* expression is common in RCC and may contribute to the pathogenesis of a subset of RCC. Further studies to reveal the mechanisms of loss of expression and its effects on the invasive behavior of renal tumor cells are required.

## 1. Introduction

RCC is the most common malignancy of the kidney and accounts for 5% and 3% of all malignancies in men and women, respectively [1]. 

The most frequent histological entity of RCC is clear cell RCC (ccRCC), representing 75% of all RCC cases [2,3]. Although there have been fundamental improvements in oncological treatment options, individualized targeted therapies for the treatment of metastatic RCC are still limited and the survival of patients with late-stage or metastatic RCC is poor [4,5]. Despite the high incidence of early-stage detection due to the wide use of imaging methods (sonography, CT, MRI) in primary care, up to one-fourth of patients have metastases at the time of initial diagnosis [6]. Thus, understanding the molecular mechanisms of the pathogenesis and progression of RCC variants for the evaluation of potential clinical benefits is of great interest.

The Cancer Genome Atlas-Kidney Renal Clear Cell Carcinoma (TCGA-KIRC) study revealed associations between the pathogenesis of RCC and various genetic and epigenetic alterations [7]. Although it has been widely assumed that genetic profiling, such as detection of tumor-specific genetic alterations including somatic mutations, polymorphisms, and chromosomal loss, might become clinically usable tools, corresponding molecular-based instruments are not yet available for RCC [8]. This might be due to the great variety of somatic mutational alterations observed in human renal tumors resulting in more or less patient-specific individual mutation spectra instead of common patterns associated with distinct clinical stages. In contrast, many other human cancers have shown frequent and consistently clinically associated epigenetic alterations such as DNA hyper- or hypomethylation. In RCC in particular, multiple studies including our own have shown that the presence of consistent systematic epigenetic changes are associated with adverse clinicopathological characteristics across different patient cohorts [2,9,10,11,12,13,14,15,16]. Hence, epigenetic alterations often associated with transcriptional silencing of the affected genes have attracted great interest, and epigenetic alterations of numerous gene candidates that might prove clinically useful have been identified by means of in silico analysis [17].

**Figure 1:**
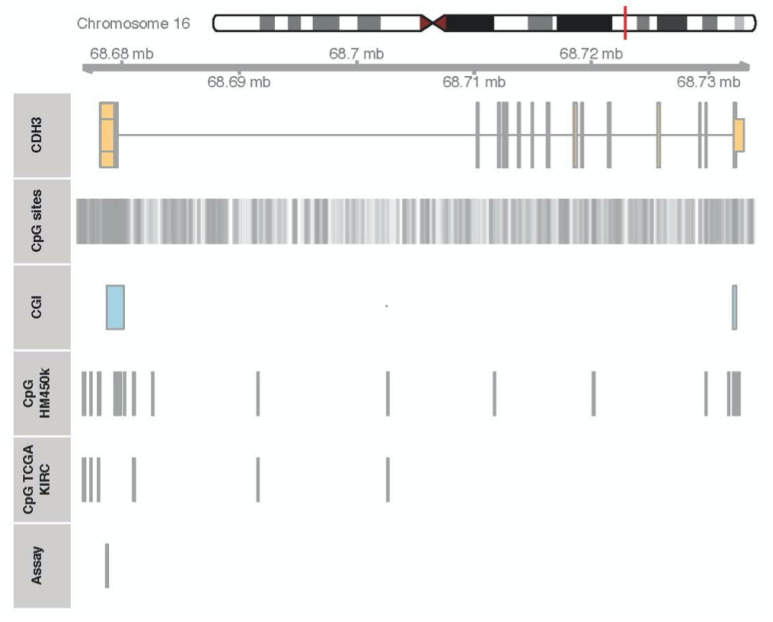
Genomic location (red line) of CDH3 exons (row CDH3: orange boxes), associated CpG islands (row CGI: blue boxes), and positions of CGI sites (row CpG sites: vertical grey lines) on chromosome 16q22.1. CpG sites annotated to the Infinium Human Methylation 450k Chip array (row CpG HM450K) and amenable to in silico analysis of TCGA-KIRC data (row CpG TCGA-KIRC) are presented. The location of the CpG sites covered by pyrosequencing and QMSP analysis is shown (row Assay: grey box). Note that CpG sites subjected to pyrosequencing and QMSP analysis are identical except for one site and, furthermore, that only one of two CpG islands annotated to *CDH3* is shown in this genomic section.

Cadherins are a class of transmembrane proteins that contribute to cell–cell adhesion in epithelial tissues—for example, in the esophagus, kidney tubules, urinary bladder, prostate, and intestines—by forming junctions and maintaining tissue integrity. Extracellular cadherin domains are known to mediate cell–cell adhesion. Cadherin 3 (CDH3, P-cadherin) has been detected in the basal layer of stratified epithelia in the placenta, epidermis, breast, and prostate [18]. Epigenetic alternations of *CDH3* with concurrent alterations in CDH3 protein expression have been correlated with adverse histopathology in a variety of cancers, such as breast, cervical, ovarian, pancreatic, gall bladder, colorectal, hepatocellular, and oral squamous cancers [18,19,20,21,22,23,24,25]. Consequently, blocking the activity of CDH3 and the associated signaling has been discussed as a novel therapeutic approach for treatment [26,27,28,29]. In other urological malignancies, including bladder and prostate cancer, *CDH3* has been shown to be negatively regulated at the genomic and transcriptional levels and associated with poor clinical outcomes [30,31]. However, how CDH3 promotes malignant transformation in different organs remains unknown. It has been shown by means of immunohistochemistry (IHC) and real-time PCR that RCCs express a complex set of cadherins, including E-cadherin (CDH1), N-cadherin (CDH2), and cadherins 4, 6, and 11, whereas no CDH3 was detected [32]. Hence, the particular role of each of these cadherins in the pathogenesis of RCC is not completely understood. The presence of two CpG islands (CGIs) within the region of chromosome 16q22.1 that includes *CDH3* (Figure 1) has led to the hypothesis that CGI hypermethylation might decrease *CDH3* expression and, in turn, facilitate the malignant transformation of renal tissue. 

In this study, we investigated whether DNA methylation, changes in mRNA and protein expression, and potential epigenetic silencing of *CDH3* occur in RCC, and correlated the presence of such changes with the histopathological and clinical characteristics of patients. We examined *CDH3* mRNA and CDH3 protein expression using real-time PCR and IHC, respectively, in normal and cancerous renal tissues. 

We demonstrate an RCC-associated loss of *CDH3* mRNA expression, the presence of DNA hypermethylation, and loss of CDH3 protein in RCC in comparison with adjacent normal tissue. siRNA-induced *CDH3* suppression resulted in altered invasion characteristics. Together, these results indicate a likely functional connection of CDH3 loss to the pathogenesis of RCC.

## 2. Results

### 2.1. Analysis of mRNA Expression in Paired Tumor and Normal Adjacent RCC Tissues

**Figure 2:**
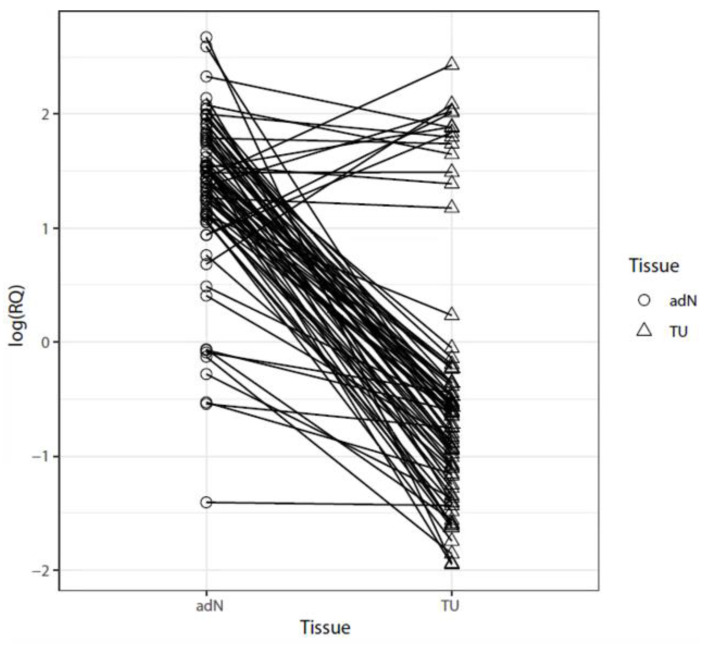
Relative quantitation of *CDH3* mRNA expression. Adjacent normal tissues (adN) and paired tumor tissues (TU) were analyzed for levels of *CDH3* mRNA expression using quantitative real-time PCR. Relative quantities of *CDH3* mRNA are presented as a log scale. Lines connect adN and TU tissues pairs. Note that a large percentage of the tumors show a pronounced tumor-specific loss of relative *CDH3* mRNA expression.

We analyzed paired tumor (TU) and normal tissues (adN; normal renal tissue excised adjacent to the tumor site) for changes in *CDH3* mRNA expression and found a clear loss of *CDH3* expression in tumors corresponding to a mean reduction of approximately 3.6-fold for relative expression values observed in TU tissues (*p* = 0.00025; Figure 2).

### 2.2. DNA Methylation of CDH3 in RCC

**Figure 3:**
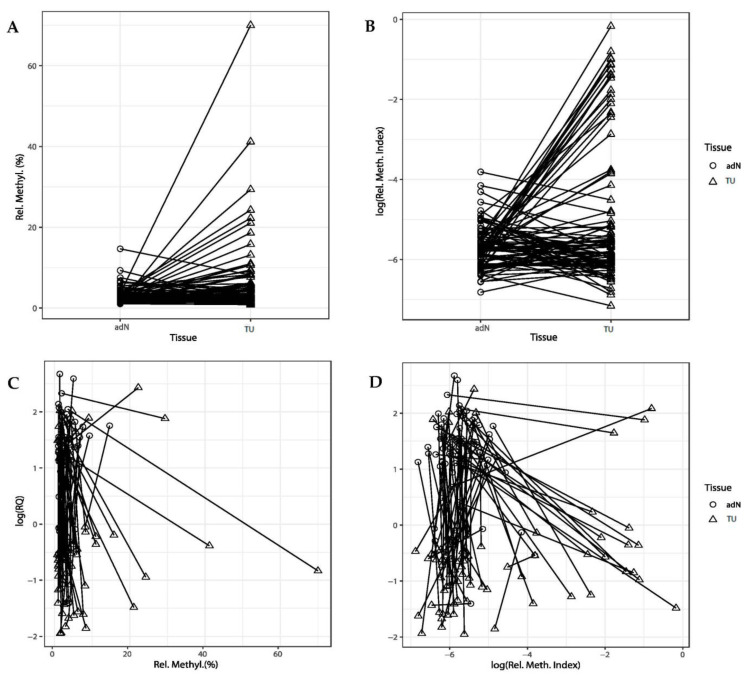
(**A**–**D**): Analysis of *CDH3* DNA methylation and expression alterations in adjacent normal tissues (adN) and paired tumor tissues (TU). Pyrosequencing (**A**) and QMSP (**B**) analysis were carried out and methylation results were compared with tissue type and the results of the relative mRNA quantitation (log scale): relative expression quantity RQ values were compared against relative methylation levels obtained by pyrosequencing (**C**) or QMSP (**D**). While tumor-specific hypermethylation was detected for both methylation detection methods in a subset of tissue pairs, epigenetic silencing, which should be detectable by the presence of a negative association of mRNA expression (RQ) and degree of methylation, appeared to be limited to a small number of tissue pairs, mostly detected in the RQ-QMSP comparison (**D**).

In order to examine whether alterations in CGI methylation of *CDH3* is associated with tumorigenesis of renal cells, we compared primary RCC and corresponding (paired) adN samples isolated from 107 patients, using both pyrosequencing to detect mean methylation levels and QMSP for relative quantitation of highly methylated sequences. In both experiments, the evaluation of individual tissue pairs showed that a fraction of tissue pairs exhibited a pronounced methylation increase in the tumor sample (Figure 3A,B). The statistical evaluation of data generated by both methylation detection methods using the two-sided paired t-test demonstrated tumor-specific hypermethylation (pyrosequencing: *p* = 0.0026, QMSP: *p* = 0.0002).

### 2.3. Comparison of DNA Methylation and CDH3 mRNA Expression 

To analyze whether *CDH3* shows epigenetic silencing, the mRNA expression and DNA methylation data from pyrosequencing were subjected to correlation analysis (Figure 3C,D). While visual examination clearly identified a small subset of individual samples demonstrating a concurrent loss of expression and high DNA methylation of *CDH3* (as detected by QMSP), no statistical correlation between expression and methylation could be found for the group of samples as a whole (*p* = 0.29; R = −0.09).

### 2.4. Association with Clinicopathological Parameters

We next evaluated the relative degree of methylation as measured by pyrosequencing in tumor samples for a possible association with clinicopathological variables. Following dichotomization (into high- and low-stage or high- and low-grade tumors), *CDH3* CGI hypermethylation was found to be associated with a higher tumor stage (low vs. high T; *p* = 0.0026; OR = 6.16 [1.99–21.60]) and higher tumor grade (low vs. high G; *p* = 0.0013, OR = 6.94 [2.26–24.41]). No significant association between methylation and the presence of lymph nodal (N0 vs. N+; *p* = 0.893; OR 1.12 [0.19–5.60]) or distant metastasis could be detected in the present cohort (M0 vs. M+; *p* = 0.297; OR 1.90 [0.56–6.43]); Table 1).

### 2.5. In Silico Analysis of Statistical Associations of CDH3 mRNA Expression and DNA Methylation Using the TCGA-KIRC Data

In order to independently verify our findings, we also queried the TCGA-KIRC database for any association of *CDH3* methylation with clinical variables including survival data. Furthermore, the mutational status and mRNA expression of key molecules of RCC carcinogenesis as well as microRNA expression were compared with *CDH3* mRNA expression and methylation state. First, we found numerous significant associations of methylation of distinct loci and adverse clinicopathological features of patients of the TCGA-KIRC cohort (Supplementary Figure S1). In addition, we found that methylation of two CpG sites of *CDH3* demonstrated significant associations with overall survival of patients in the Cox regression analysis (Supplementary Figure S2). A corresponding analysis for the expression of key RCC molecules including *VHL*, *SETD2*, *PBRM1*, *HIF1A*, and others, supplemented by *CDH1* and *CDH3* expression data, only demonstrated a significant contribution for *CDH3* expression besides age (Supplementary Figure S3). Furthermore, we addressed the question of whether mutational status or expression alterations of key RCC molecules show associations with altered mRNA expression or methylation of *CDH3*. A Pearson’s correlation analysis revealed that the mutational status of *VHL* was not associated with methylation of any of the *CDH3* CpG sites (Supplementary Figure S4). In contrast, a weak but significant correlation between *SETD2* mutations and *CDH3* methylation was indicated for six out of the nine CpG sites. Interestingly, the corresponding comparison of mRNA expression and methylation demonstrated a significant negative correlation for *SETD2* expression and two CpG sites of *CDH3* (Supplementary Figure S5). Finally, we examined whether expression of specific microRNAs show association with mRNA expression of RCC key molecules expanded by *CDH3* and *CDH1*. Of note, we found a comparatively strong negative association between hsa-mir-204 and *CDH3* expression (Supplementary Figure S6). Moreover hsa-mir-192, hsa-mir-194-1, and hsa-mir-194-2 showed significant negative associations with the expression of *CHD3*, *HIF1A*, *PBRM1*, *SETD2*, and *VHL*.

### 2.6. Immunohistochemical Analysis of CDH3 Protein Expression 

To characterize CDH3 protein expression and the distribution of CDH3 immunopositivity in renal tissues, we conducted an IHC analysis. We found that 10/20 tumors and 19/20 normal renal tissues demonstrated immunopositivity in at least 60% of cells (*p* < 0.001, chi-square test).

**Figure 4:**
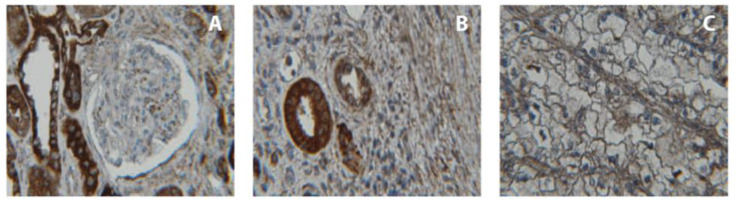
(**A**–**C**): Immunohistochemical analysis of CDH3 protein expression in normal noncancerous (**A**) and cancerous renal tissue ((**B**): invasion zone; (**C**): tumor; magnification: 40×). Control staining omitting the primary antibodies did not reveal positivity in any of the histological components of the kidney Note that, in normal tissue, CDH3 was mainly present in epithelial cells lining the tubules and collecting ducts, and that immunopositivity disappeared in areas affected by tumor growth.

The immunopositivity in normal tissues was located in the cytoplasm of epithelial cells lining the distal tubules and collecting ducts, whereas glomerular structures appeared completely free of staining. The proximal tubules demonstrated less immunoreactivity (Figure 4).

**Figure 5:**
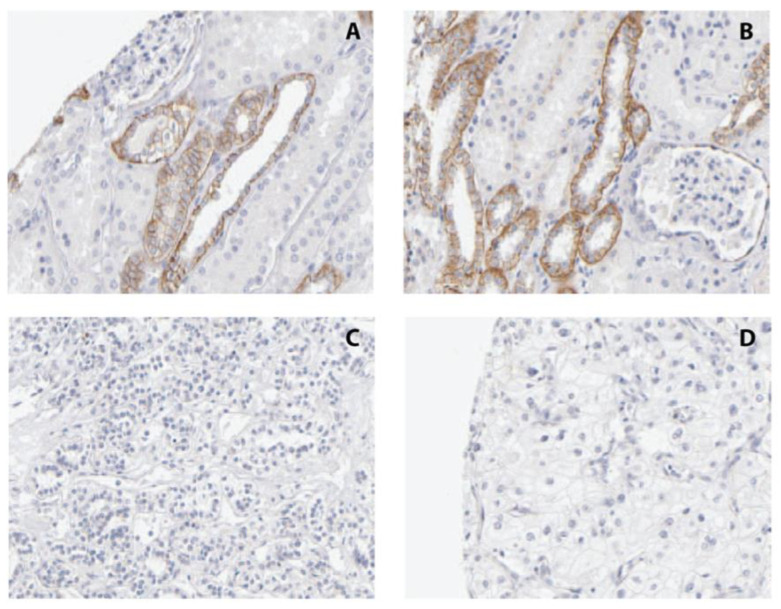
(**A**–**D**): Immunohistochemical analysis of CDH3 protein expression as presented by the Human Protein Atlas. Immunopositivity for the CDH3 protein was detected in the epithelial layers of presumed distal tubules and collecting ducts of noncancerous renal tissues of a 56-year-old female (**A**) and a 28-year-old male (**B**). Loss of CDH3 immunopositivity was observed in RCC samples taken from a 76-year-old female (**C**) and a 57-year-old male (**D**; magnification: 40×).

In addition, we examined the immunohistochemical data published by the Human Protein Atlas [33]. There, using antibody HPA001767, 10/11 (90.91%) RCC tissues displayed no signal, while 1/11 (9.09%) showed low positivity in histological areas affected by cancer lesions. In sections stained using the antibody CAB002487, 11/12 (91.67%) RCC tissues showed no signal, whereas 1/12 (8.33%) had medium positivity. In contrast, pronounced immunopositivity could be seen in normal renal cortex in collecting ducts and distal tubules, with medium staining intensity in 3/3 samples (100%) stained using HPA001767 and with high intensity in cells in tubules in 3/3 samples (100%) stained with CAB002487 (Figure 5). Applying Fisher exact statistics to both immunohistochemical analyses, we found that the null hypothesis of no staining differences between normal and tumor tissues was rejected for both antibodies (*p* < 0.02; *p* < 0.01).

### 2.7. Epigenetic and Functional Alterations of CDH3 in Cell Line Tumor Models

**Figure 6:**
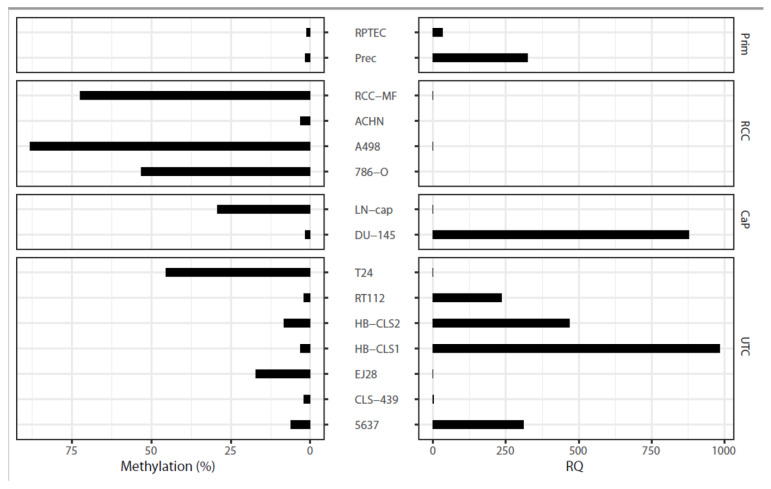
Comparison of results obtained by pyrosequencing for quantitation of the relative degree of *CDH3* DNA methylation (**left**) and relative mRNA levels measured by real-time PCR (**right**) in various normal cell and tumor cell models. Healthy renal tissue (Prim), renal cell cancer tumor cells (RCC), prostate cancer cells (CaP), and urothelial carcinoma tumor cells (UTC) were analyzed. The majority of the cell lines revealed an inverse relationship between the relative degrees of *CDH3* DNA methylation and *CDH3* mRNA expression.

**Figure 7:**
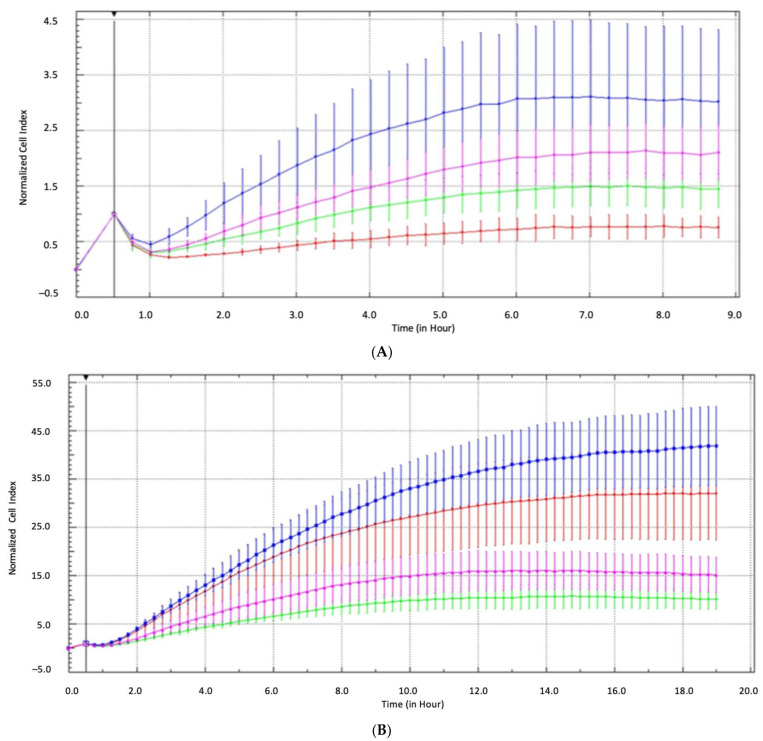
(**A**,**B**): Effects of siRNA-induced suppression of CDH3 expression on the Matrigel-transiting ability of cells as a surrogate for invasive potential of the A498 (**A**) and 786-O (**B**) RCC cell lines. Real-time impedance-based measurements showing normalize cell index vs. time in hours of RCC cell lines in the Matrigel invasion assay following pretreatment of cells for re-expression with 0.125 µM or 0.5 µM 5-AZA and subsequent application of siRNA for sequential re-expression and suppression of CDH3. Control runs were carried out for both of the 5-AZA concentrations with 25 nM of each mock siRNA (on Target plus control, red curves for 0.125 µM and green curves for 0.5 M AZA pre-incubations) and compared with cells treated with 25 nM of each CDH3 siRNA (blue curves for 0.125 µM and pink curves for 0.5 µM 5-AZA pre-incubations). Impedance is shown as normalized cell index versus time (seconds) and reflects the migration of cells from a nutrient- and growth factor-deficient medium through the Matrigel barrier and growth on a microelectrode in the growth medium-containing second chamber. Note that a comparison of the blue and pink siRNA curves vs. the corresponding red and green control curves indicates increased cellular mobility through the Matrigel barrier for cells subjected to CDH3 expression suppression.

To gain additional information about the relevance of *CDH3* alterations in RCC tumorigenesis, we analyzed DNA methylation, mRNA expression, and the effect of siRNA-induced suppression of *CDH3* expression on cell invasiveness in various human cancer cell line models. Using pyrosequencing to quantify the relative degree of DNA methylation, we found that *CDH3* showed a substantial degree of methylation in the majority of kidney cancer cell lines, including A498 (relative methylation: 90%), 786-O (55%), RCC-GS (90%), and RCC-MF (65%); in one of two prostate cancer cell lines (LN-cap); and in two of seven bladder cancer cell lines (T24 and EJ28); while low methylation levels were found in primary cells used as normal tissue surrogate models (Figure 6). Of note, the quantitative mRNA expression analysis using real-time PCR revealed largely reciprocal values for *CDH3* expression when compared with the degree of relative methylation. A Spearman rank correlation analysis of the 15 cell lines with available data on DNA methylation and mRNA expression demonstrated a coefficient of correlation R = −0.58 (*p* = 0.023), indicating that DNA methylation-related epigenetic silencing of *CDH3* expression could occur in various human cancers. In order to preliminarily evaluate whether, similar to the gene family member *CDH1*, functional changes associated with cell adhesion and/or mobility might be relevant, we carried out an impedance-based real-time analysis of cell invasiveness following siRNA-induced suppression of *CDH3* expression. Considering that none of the typical RCC cell lines A498, 786-O, or RCC-MF demonstrated endogenous *CDH3* expression (Figure 6), we first cultured A498 and 786-O cells in the presence of 5-aza-2’desoxycytidine (5-AZA) in order to reconstitute it. Following Western blotting confirmation of endogenous re-expression of *CDH3* (Supplementary Figure S7), we confirmed the effect of siRNA-induced *CDH3* knockdown by analyzing each cell line in triplicate by real-time impedance measurements compared with siRNA mock controls. In 3/4 cases, a consistent increase in the ability to pass the Matrigel barrier in a time window of 20 h was observed (Figure 7A,B). Additionally, we examined the effects of siRNA-induced suppression of *CDH1 CDH3* expression in a bladder cancer cell line model 5637, which, unlike the RCC cell line used in our study, shows substantial endogenous *CDH3* expression. Here, siRNA-induced suppression of *CDH3* resulted in increased invasive characteristics in this model, while this was not the case for *CDH1* (Supplementary Figure S8).

The Western blotting results, in the case of A498 with both concentrations of 5-AZA, confirmed a pronounced re-expression of *CDH3* and a corresponding increase in cellular mobility in the Matrigel invasion assay could be observed. For 786-O cells pre-treated with 0.125 µm 5-AZA, a particularly large mobility difference was obtained in the real-time mobility analysis, although Western blotting indicated limited protein-re-expression (Supplementary Figure S7).

## 3. Discussion

In this study, we found changes in *CDH3* mRNA expression, DNA methylation, and protein expression in RCC that were associated with histopathological and clinical characteristics of patients, while siRNA-induced suppression of *CDH3* expression altered RCC cell invasiveness.

A considerable number of studies have been carried out linking epigenetic alterations such as DNA methylation in genes with carcinogenesis in RCC. Cadherins are a family of proteins involved in cellular differentiation, cell adhesion, and maintenance of the integrity of adult tissue including multilayered epithelia. In particular, alterations in the family members *CDH1* and *CDH3* have been reported to be associated with malignant transformation of various tissues and the progression of a variety of cancers, such as HCC, adenocarcinoma of the gall bladder, pancreas, colon and rectum, breast, urinary bladder, and prostate [21,26,27,28,29,30,31]. CGI hypermethylation of *CDH1* is associated with loss of mRNA and protein expression, accompanied by a loss of cellular differentiation, progressive disease, and in some cases, metastasis in RCC [34,35]. While the expression (reduced in some cell lines) of a number of cadherins, including CDH1, has been detected in RCC, CDH3 alterations have not been described. The expression characteristics of cadherins in RCC appear to be complex [32]. Moreover, hypermethylation of *CDH1* was mainly observed in ccRCC, the most common subtype of RCC [12]. While this, overall, suggests that changes in cadherin gene methylation and expression affect RCC tumor biology, the role of the chromosomal neighbor *CDH3* is still not clear. 

Our quantitative mRNA expression analysis first demonstrated a statistically significant loss of *CDH3* expression affecting three-quarters of the paired tumoral and normal tissues. Moreover, although an overall difference in tumor DNA hypermethylation was observed in the paired tissue comparison, visual analysis revealed that in both cases, only a subset of tissue pairs exhibited a tumor-specific increase in DNA methylation.

To gain statistical evidence for the presence of possible epigenetic silencing, we compared DNA methylation and mRNA expression using both of the methylation datasets but did not find a significant relationship. However, visual inspection of data pairs again demonstrated heterogeneity, as only a subset of tumors showed a negative association between DNA methylation and mRNA expression, indicating the need to enlarge the study group to determine whether these differences are statistically significant. 

Considering that a large percentage of tumors obviously showed a loss of *CDH3* expression independent of DNA methylation, we speculated about the presence of specific mutational and/or mRNA and/or microRNA alterations as an additional layer of expression control. Querying the TCGA-KIRC data, we found that methylation of *CDH3* has significant but comparatively weak correlations with *SETD2* mutations (Supplementary Figure S4), but it seems to be largely independent from VHL mutations and *HIF1alpha* expression (Supplementary Figures S4 and S5). On the other hand, the expression of *CDH3* showed significant and comparatively strong negative correlations with a number of microRNAs (Supplementary Figure S6) including hsa-miR-204, which is already known to show a strong association with invasive behaviors of tumor cells [36]. Notably, the expression of a substantial part of key RCC players *(VHL*, *PBRM1*, *HIF1A*) also showed a negative correlation with has-miR-204 expression and the candidate microRNAs hsa-miR-192, hsa-miR-194-1, and -2. Overall, the in silico analysis of the TCGA-KIRC data provided a hypothetical explanation for the methylation-independent epigenetically induced changes in *CDH3* expression, thus serving as a possible starting point for subsequent targeted functional analyses.

Our comparison of DNA methylation and clinicopathological variables of patients demonstrated that increased methylation of *CDH3* is associated with higher tumor stages and grades of differentiation, while no statistical association was seen between *CDH3* methylation levels and the state of distant or lymph node metastasis. The in silico analyses of the TCGA-KIRC database also revealed significant associations between the methylation of distinct loci and the stage, grade, and state of distant metastasis of patients, thus confirming and extending our findings of a correlation between methylation and clinical metastasis (Supplementary Figure S1). Furthermore, the in silico multivariate Cox proportional hazard regression analyses of overall survival of the TCGA-KIRC cohort revealed the methylation of two CpG sites with a potentially high increased risk of death (Supplementary Figure S2). A corresponding analysis for mRNA expression and survival including the key RCC molecules expanded by the adhesion molecules CDH1 and CDH3 revealed only *CDH3* mRNA expression and age as statistically significant predictors for survival (Supplementary Figure S3).

The pyrosequencing analysis of commercially available cell lines derived from human kidney cancer demonstrated a high degree of methylation in the majority of the cell lines, indicating that *CDH3* CGI methylation might be a relevant event in the development of RCC. This is supported by the observation that significant hypermethylation of *CDH3* for both average and dense patterns of methylation was found in our analysis, but apparently not in papRCC [12]. These findings correspond to those of earlier studies reporting that the methylation of *CDH3* and a related reduction or complete loss of protein expression is associated with increased risk of HCC and also correlates with tumor staging and proliferation [28]. Comparable results have been described for bladder cancer where the loss of the CDH3 protein signal was associated with muscle invasiveness, high grade (G3) tumors, nodal extension, and poor clinical outcomes and prognoses with respect to long-term and overall survival [30]. In colorectal, pancreatic, and breast cancers, and in cell/adenosquamous carcinoma of the gallbladder, increased expression of *CDH3* was significantly associated with progression markers such as cell differentiation, tumor size, lymph node infiltration, and outcome of surgical intervention, and a reduction in overall survival, thus indicating that *CDH3* appears to act in a context-dependent manner as either a tumor suppressive or oncogenic factor in different carcinomas [21,22,27,37].

Our immunohistochemical examination of CDH3 expression revealed a significant loss of its signal in cancerous tissues, while immunopositivity was seen in noncancerous areas adjacent to the tumor site and in specimens from tumor-free kidneys. The localization of immunopositivity in epithelial cells in the distal part of the tubules and the collecting duct matches the immunostaining in the Human Protein Atlas. These findings might also support the hypothesis that ccRCC does not develop exclusively from epithelial cells of the proximal tubule, but also from distal parts of the nephron [38].

Our preliminary functional analyses measuring the effects of suppressing CDH3 expression following unspecific endogenous re-expression induced by exposure of the various cancer cell models to 5-AZA indicated that changes in CDH3 expression might be associated with changes in cell invasiveness, as two RCC and one bladder cancer cell line models exhibited altered mobility when migrating through a Matrigel barrier in the real-time impedance analysis. Considering that the renal cancer cell line models tested so far displayed high methylation and low CDH3 expression, which is in contrast to the bladder cancer cell line model showing substantial endogenous expression, our functional analyses using unspecific re-expression of CDH3 are nevertheless limited. Improvements such as the use of specific ccRCC cell line models combined with specific re-expression of CDH3 are required to further confirm these data. On the other hand, possible functional modulations of cell invasiveness or adhesion have been associated with changes in CDH1 and CDH3 expression in other cancers [20,39].

Recently, we defined a methylation signature indicating the metastatic status of renal tissues with high diagnostic efficiency [9]. Thus, additional information about methylation alterations in *CDH3* in RCC might also be useful in extending and/or improving methylation signatures identifying aggressive tumors. This analysis could be a potential noninvasive preoperative risk stratification tool for a future adjuvant treatment such as the PD-1 checkpoint inhibitor pembrolizumab, which has been shown to improve disease-free survival among patients who underwent nephrectomy and were at high risk for recurrence [40].

Taking into account the substantial number of genes that are known to exhibit both alterations in DNA methylation and functional alterations, our findings indicating a possible functional link of *CDH3* methylation alterations and cell invasiveness might also contribute to defining new potential therapeutic targets for future pharmaceutical interventions [17]. In addition, provided that the functional involvement of *CDH3* in RCC tumorigenesis can be confirmed in future analyses, these data further underscore the potential relevance of approaches aimed at the therapeutic benefit of demethylation medication [41].

## 4. Material and Methods

### 4.1. Tumor Cell Lines

Renal proximal tubular epithelial cells (RPTECs) were obtained from Lonza AG (Basel, Switzerland). Cancer cell lines from the kidney (ACHN, A498, RCC-GS, RCC-HS, RCC-MF, 786-O), urinary bladder (RT112, CLS-439, HB-CLS1, HB-CLS2, EJ28, 5637, T24), and prostate (DU-145, LN-cap) were purchased from Cell Line Services (Eppelheim, Germany). The cells were cultured and maintained according to the manufacturers’ instructions and were at passage 18 at the beginning of the experiments.

### 4.2. Tissue Specimens

A quantitative analysis was conducted using 107 (total of 214) paired RCC specimens (75 ccRCC, 22 papillary, 3 chromophobe, 4 unclassified, and 3 mixed RCC) and a subgroup of 81 (total of 162) paired samples of TU and adN.

Tissue samples were obtained from patients who underwent partial or radical nephrectomy. The specimens were immediately shock-frozen in liquid nitrogen and stored at −80 °C. Tumor stages were assessed according to the UICC 2002 issue of the TNM system. Nuclear grading was based on the Fuhrman grading system. Histological subtypes were assessed according to the consensus classification of RCC. Localized RCC was defined as ≤pT2 with no positive lymph nodes (N0) or metastasis (M0), G1 or G1 to G2. Advanced RCC was characterized as ≥pT3 and/or positive lymph nodes (N+) and/or metastasis (M+), and G2 to G3 or G3. The clinical and histopathological characteristics of the tissue donors are summarized in Table 2 [2,42,43,44].

### 4.3. Immunohistochemical and Western Blotting Analysis of CDH3 Protein Expression

IHC was carried out using paraffin-embedded tissue microarrays of renal tissues representing 20 samples each of normal, invasion front, and tumor tissue, as described previously [2,42,43,44]. Briefly, the immunostaining was performed following unmasking and application of an avidin/biotin blocking kit (Vector Laboratories, Burlingame, CA, USA). For in silico analyses of the tissue expression of CDH3 in normal kidney and RCC, we queried the Human Protein Atlas (www.proteinatlas.org, accessed on 10 November 2023) data obtained from the use of the HPA001767 and CAB002487 antibodies [33].

For antigen detection, a purified mouse anti-P-Cadherin antibody (1:100; BD Biosciences, Heidelberg, Germany), a biotinylated horse anti-mouse IgG (1:200; Vector Laboratories, Burlingame, CA, USA), and the Vectastain Elite ABC Kit and DAB Peroxidase Substrate Kit (Vector Laboratories) were used. A negative control omitting the primary antibody was included. Western blotting was carried out as described previously [42] but with the use of the anti-P-Cadherin antibody (BD Biosciences) as the primary antibody for antigen detection.

### 4.4. Isolation of RNA, cDNA Synthesis, and Real-Time PCR Quantitation

The extraction of total RNA, cDNA synthesis, and real-time PCR were carried out as described previously but with the use of TaqMan assays for CDH3 (Hs00999915_m1; Thermo Fisher Scientific, Waltham, MA, USA) for the detection of the target sequence and HPRT1 (Hs01003267_m1), PPIA (Hs99999904_m1), and RPL13AP5-(Hs03043885_g1) was used as the endogenous controls [42]. For the calculation of relative mRNA quantities, the SDS 2.3 Manager data assist v2.0 software and the delta delta Ct method were applied [45,46].

### 4.5. DNA Extraction, Bisulfite Conversion, and Methylation Analyses

The isolation of genomic DNA from frozen sections of malignant and normal renal tissue and bisulfite conversion of DNA have been reported previously [10]. Methylation analyses were carried out using pyrosequencing and quantitative methylation-specific PCR (QMSP). The genomic targets of both assays are illustrated in Figure 1. The pyrosequencing analysis of *CDH3* CGI methylation was technically conducted as described previously [47]. Briefly, forward (5′-GGGGTTGAGTTTTTTGGGGTTAAG-3′), reverse (5′-CCTCCCCCCTCCAAAATCACTTCA-3′), and sequencing (5′-GGATTTAGTTTTTTTTATTTTTTGT-3′) primers were designed using PyroMark Assay Design 2.0 software (Qiagen, Hilden, Germany). PCR, purification of biotinylated PCR products, preparation of single-stranded DNA, annealing, and pyrosequencing were carried out using the PyroGold SQA kit and a PyroMark Q24 System (Qiagen, Hilden, Germany) according to the manufacturer’s protocol. To analyze the sequence TTTYGGGYGTAGGTTTGTTGGTTGTAGTGYGYGGTTTTYGAGTYGTGTYGGGYGGTTTTTAGGGAGGTTGAAGTGATT, CpG sites at the genomic base positions 68, 679, 530, −34, −56, −58, −65, −70, −75, and –79 on chromosome 16 were evaluated using methylation PyroMark Q24 software (Supplementary Figure S9 for exemplary primary data). An independent methylation analysis of additional CpG sites of *CDH3* was performed using QMSP as described previously for a different target gene [11]. The primer design for the *CDH3* QMSP was carried out using Beacon Designer8 software (Premier Biosoft, San Francisco, CA, USA), which specified the forward primer (5′-ACCCTCGAACGCAAATTTACTAA-3′), the reverse primer (5′-TTTTAGCGTTTGGTCGGGTTT-3′), and the fluorometric probe (5′-FAM-CGACCTCCGAACCGTACCGAACGATC-BHQ 3′) oligonucleotides [48]. The QMSP covered a total of 9 CpG sites located on chromosome 16 at 68, 679, 530, −534, −558, −565, −570, −575, −579, −634, and −642. 

### 4.6. Real-Time Impedance Analysis of Cell Invasiveness

The analysis of possible changes in the cell invasiveness of the RCC cell line tumor models A498 and 786-O and of the bladder cancer cell line model 5637 induced by siRNA suppression of *CDH3* expression was carried out by real-time impedance measurements of the tumor cells’ ability to pass from a nutrient- and growth factor-deficient medium through a Matrigel barrier into a normal growth medium chamber as described previously [11]. Briefly, considering that all RCC cell line models investigated exhibited a high degree of *CDH3* methylation associated with low mRNA expression, we first conducted undirected demethylation by culturing the cells in the presence of 5-AZA and performed Western blotting analysis to control for the endogenous re-expression of CDH3 protein.

The transfection of cells was then carried out with a mixture of 25 nM each of four ON-TARGETplus siRNAs (Thermo Fisher Scientific, Waltham, MA, USA) for knockdown of *CDH3* expression (Target) or the control plasmid (TargetPlus, siRNA-negative) as described previously [11]. The siRNA sequences were 5′-UGAAUCAGCUCAAGUCUAA-3′, 5′-GUGACAACGUCUUCUACUA-3′, 5′-GAAAUCGGCAACUUUAUAA-3′, and 5′-GAGGGUGUCUUCGCUGUAG-3′.

All real-time impedance cellular invasiveness experiments used 30,000 cells per well and a Matrigel concentration of 1.5% and were performed in triplicate. 

### 4.7. Statistical Analysis

To compare the degree of DNA methylation in RCC and paired adN, the two-tailed paired *t*-test was applied. The relative degree of methylation was calculated according to Weisenberger [48,49]. Possible associations of the degree of methylation and clinicopathological variables of patients such as localized vs. advanced disease, state of lymph node metastasis (N0 vs. N+), absent and present distant metastasis (M0 vs. M+), and tumor differentiation (low grade G1 and G1-2 vs. high grade G2-3 and G3) were analyzed using logistic regression analysis. R statistical software (version 3.6) was used to perform all statistical analyses. *p* values ≤ 0.05 were considered to be statistically significant. In silico analyses were performed by querying the TCGA-KIRC database [7]. The association of relative methylation and clinicopathological parameters was carried out by logistic regression analysis as described above. A Pearson’s correlation analysis was carried out for the detection of associations between mutational status, mRNA expression levels, and degree of CpG-specific methylation. Multivariate Cox proportional hazard models were generated to estimate the hazard ratios in the overall survival analysis. Adjustments for multiple statistical testing used the Benjamini–Hochberg correction.

## 5. Conclusions

This study revealed *CDH3* alterations at the DNA, mRNA, protein, and functional levels and therefore underlines the possible relevance of this gene in the tumorigenesis and progression of RCC. Thus, the necessity for a further, more detailed analysis of *CDH3* function in RCC is underscored.

## Figures and Tables

**Table 1:** Statistical association of *CDH3* methylation and clinicopathological parameters.

*CDH3* Methylation	OR (95% CI)	*p* Value
ccRCC vs. papRCC	11.64 (2.47–72.30)	0.0041
Tumor stage (low vs. high T *)		
All RCC	6.16 (1.99–21.60)	0.0026
ccRCC	3.49 (0.98–13.90)	0.061
Lymph node status (N0 vs. N)		
All RCC	1.12 (0.19–5.60)	0.893
ccRCC	0.98 (0.09–8.14)	0.983
Metastasis (M0 vs. M)		
All RCC	1.90 (0.56–6.43)	0.297
ccRCC	1.00 (0.24–4.04)	0.995
Differentiation (low vs. high G **)		
All RCC	5.59 (1.41–24.86)	0.017
ccRCC	4.67 (0.98–25.68)	0.059
State of disease (loc. vs. adv. ***)		
All RCC	6.94 (2.26–24.41)	0.0013
ccRCC	4.31 (1.20–17.94)	0.032

Group comparisons were carried using logistic regression analysis following dichotomization if necessary. * Low (≤pT2) vs. high (≥pT3) tumor stage; ** low (G1 or G1–2) vs. high (G2–3 or G3) differentiation; *** localized (≤pT2, N0, M0, G1 or G1–2) vs. advanced (≥pT3 and/or N+, M+ or G2–3 or G3) state of disease; loc = local, adv. = advanced.

**Table 2:** Tumor cohort characteristics with paired adN (adjacent normal renal tissue excised adjacent to the tumor site).

Total Cases, n (%)	107 (100%)
Histology	
ccRCC	75 (70.1%)
papRCC	22 (20.6%)
Chromophobe	3 (2.8%)
Mixed histology	3 (2.8%)
Other	4 (3.7%)
Sex	
Female	40 (37.4%)
Male	67 (62.6%)
Age, median (range)	64 years (35–91)
Synchronous lymph node metastasis	
N0	95 (88.8%)
N+	12 (11.2%)
Synchronous distant metastasis	
M0	85 (79.4%)
M+	22 (20.6%)
T-classification	
pT1	59 (55.14%)
pT2	7 (6.5)
pT3	39 (36.45%)
pT4	1 (0.93%)
na	1 (0.93%)
Differentiation	
G1	20 (18.7%)
G1–G2	14 (13.1%)
G2	56 (52.3%)
G2–G3	6 (5.6%)
G3	11 (10.3%)
State of disease	
Localized (≤pT2, N0, M0, G1 or G1–2)	56 (52.3%)
Advanced (≥pT3 and/or N+, M+ or G2–3 or G3)	50 (46.73%)
NA	1 (0.9%)

## Data Availability

Anonymized datasets used and/or analyzed during the current study are available from the corresponding author upon reasonable request as part of data is subject to regulation by the German General Data Protection Regulation (Art.5 DSGVO). All data used for in silico analysis can be obtained from the Genomic Data Commons Data Portal (https://gdc.cancer.gov/access-data/gdc-data-portal, accessed on 10 November 2023).

## Supplementary Materials

The following supporting information can be downloaded at: https://www.mdpi.com/article/10.3390/ijms242216476/s1.
