# Supplementary material for: Alteration of Cadherin 3 Expression and DNA Methylation in Association with Aggressive Renal Cell Carcinoma
Source: Int J Mol Sci. 2023 Nov 18;24(22):16476. doi: 10.3390/ijms242216476 (PMC10670999; doi:10.3390/ijms242216476)
Supplement: Supplementary file 1 [file ijms-24-16476-s001.zip › ijms-2664271-supplementary.pdf]

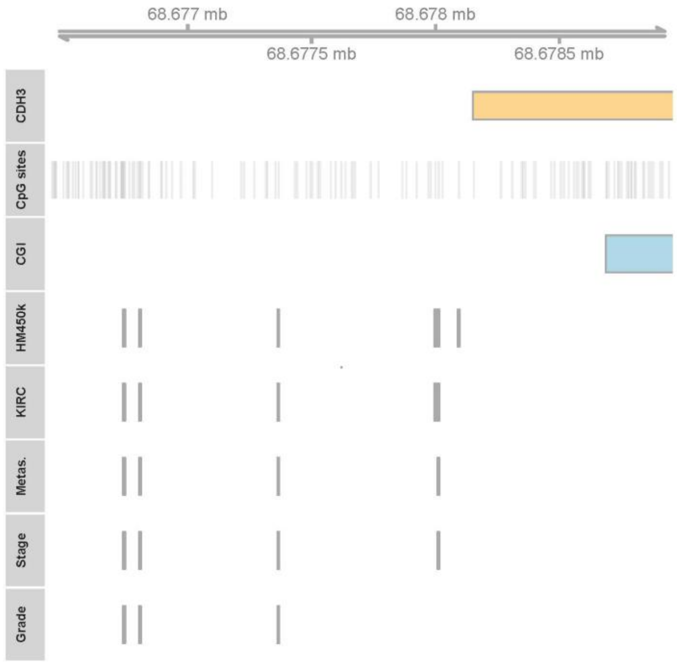

Figure S1. Location of CDH3 exons, CpG islands (CGI, blue boxes), and related CGIs on chromosome 16q22.1. Vertical grey lines represent CpG sites annotated to the Infinium Human Methylation 450k Chip array (row CpG HM450K), amenable to in silico analysis of TCGA-KIRC data (row KIRC), and showing a statistically significant association with the state of distant metastasis (Metas.), or low (≤pT2) or high (≥pT3) stage tumors (Stage), and low (G1 or G1–2) or high grade (G2–3 or G3) tumors (Grade) in logistic regression analysis (all $p < 0.05$).

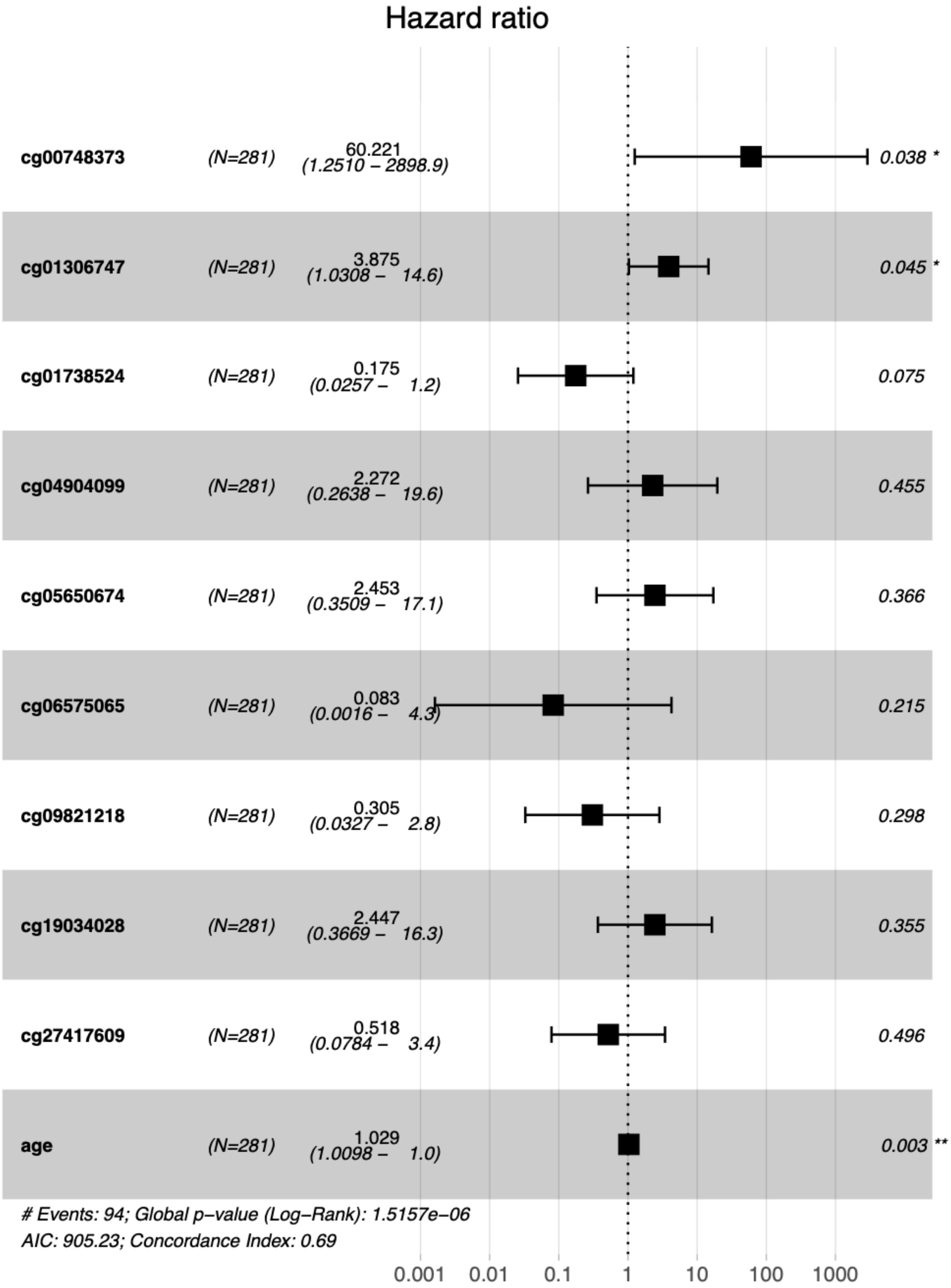

Figure S2. Forest plot of multivariate Cox proportional hazard regression analysis of overall survival of the TCGA-KIRC cohort and DNA methylation of *CDH3*. Hazard ratios and confidence intervals are shown. Note that two CpG sites show a potential strong association with increased risk for death (hazard ratio > 3.9).

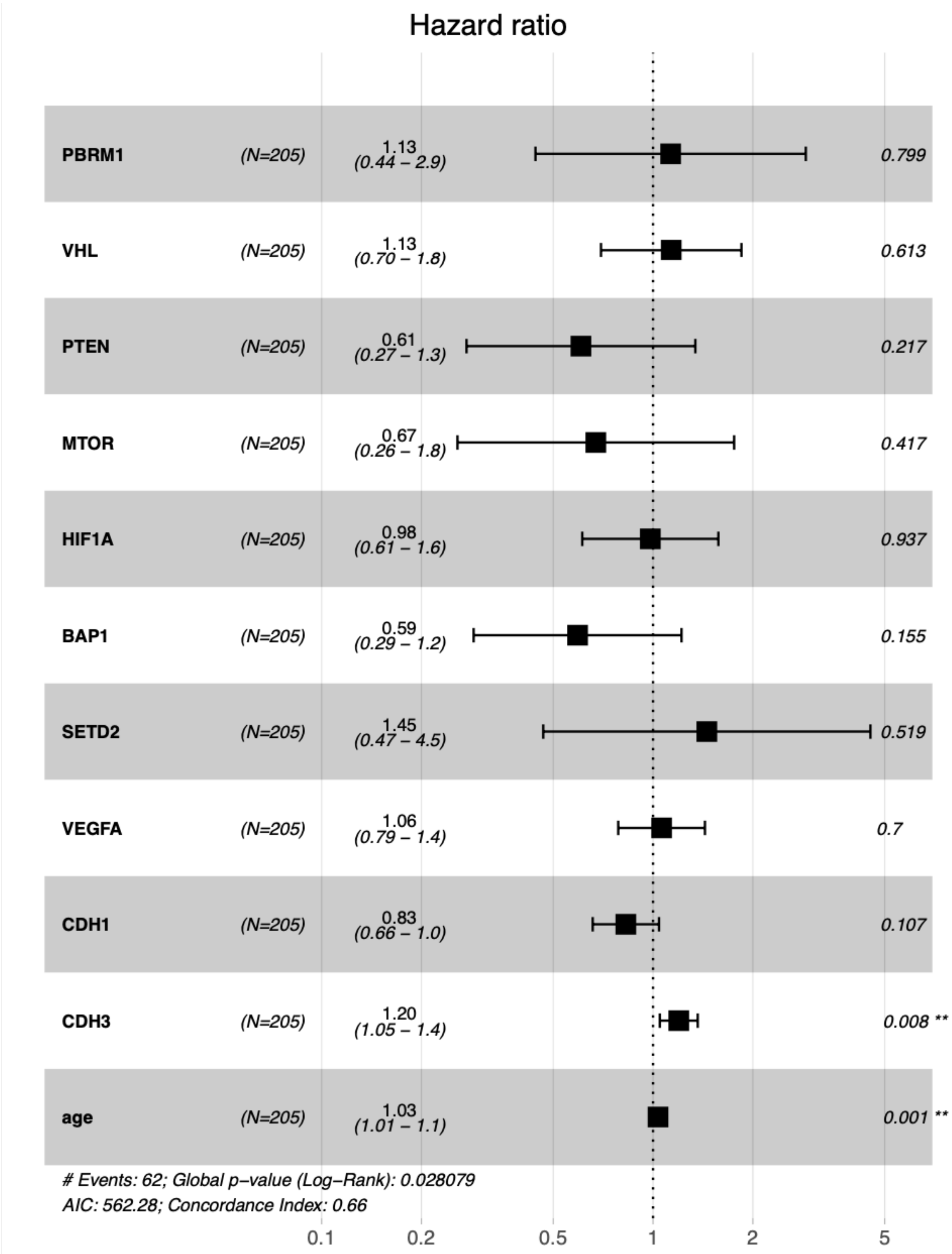

Figure S3. Forest plot of multivariate Cox proportional hazard regression analysis of overall survival of the TCGA-KIRC cohort and mRNA expression of key RCC molecules including the adhesion molecules CDH3 and CDH1. Hazard ratios and confidence intervals are shown. Note that only the expression of CDH3 and age reached statistical significance for a contribution to the survival model.

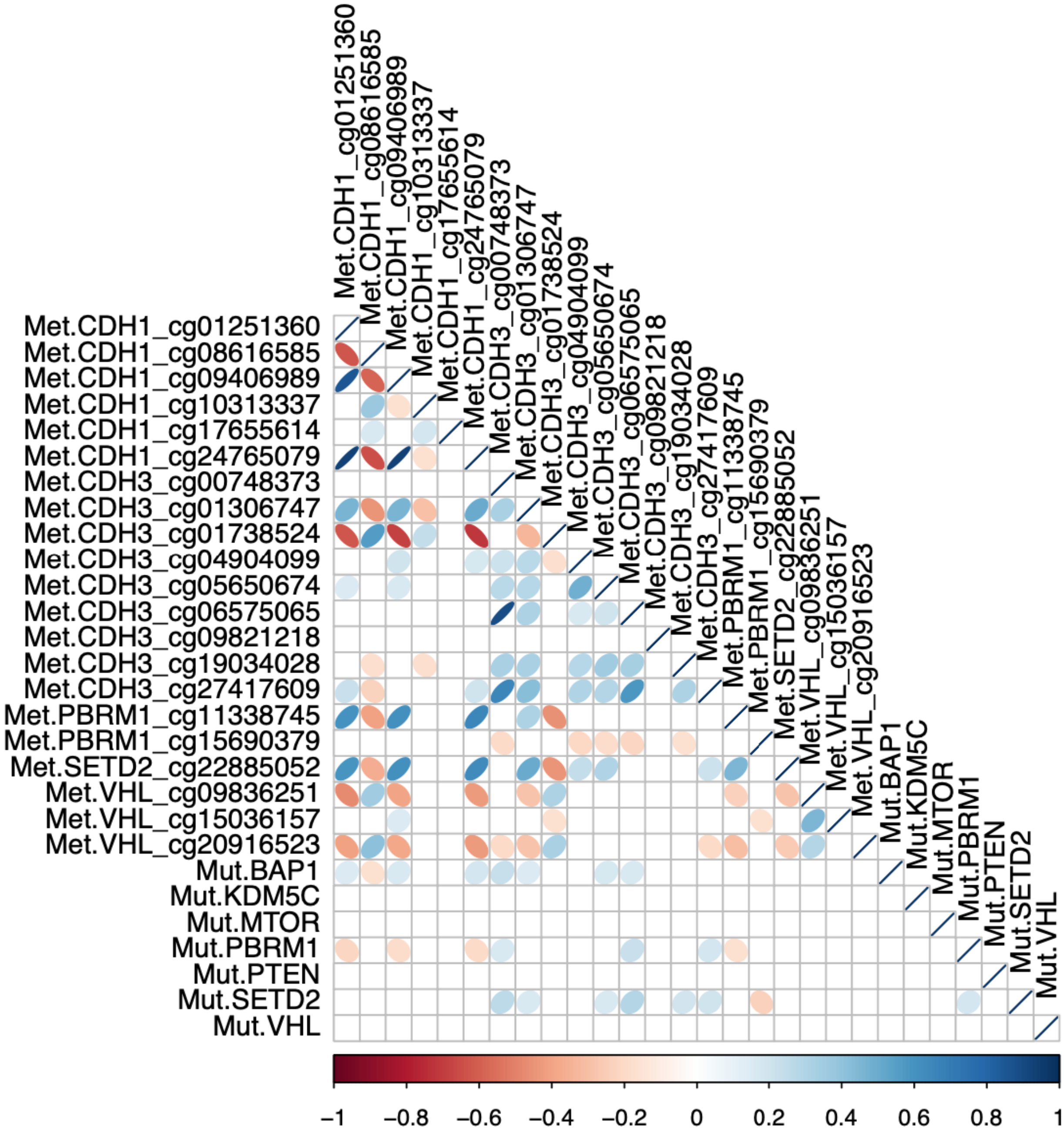

Figure S4. Presentation of Pearson's correlation analysis of associations of mutations in the most frequently mutated genes (prefix "Mut.") in ccRCC and CpG site-specific DNA methylation (prefix "Met.") of CDH3 according to the TCGA-KIRC study data. Correlations exhibiting $p < 0.05$ following Benjamini–Hochberg correction for multiple testing are presented as blue and red ellipsoids indicating positive and negative coefficients of correlation R according to the color-coding bar. The size of R is additionally indicated by the width of the ellipsoids, indicating perfect correlation of R = 1 as angle bisecting lines with a gradient of 1 or –1. Note that mutations in VHL do not show any association with methylation of CDH3. In contrast, there seem to be weak correlations between SETD2 mutations and CHD3 methylation as well as PBRM1 mutations and CDH1 methylation.

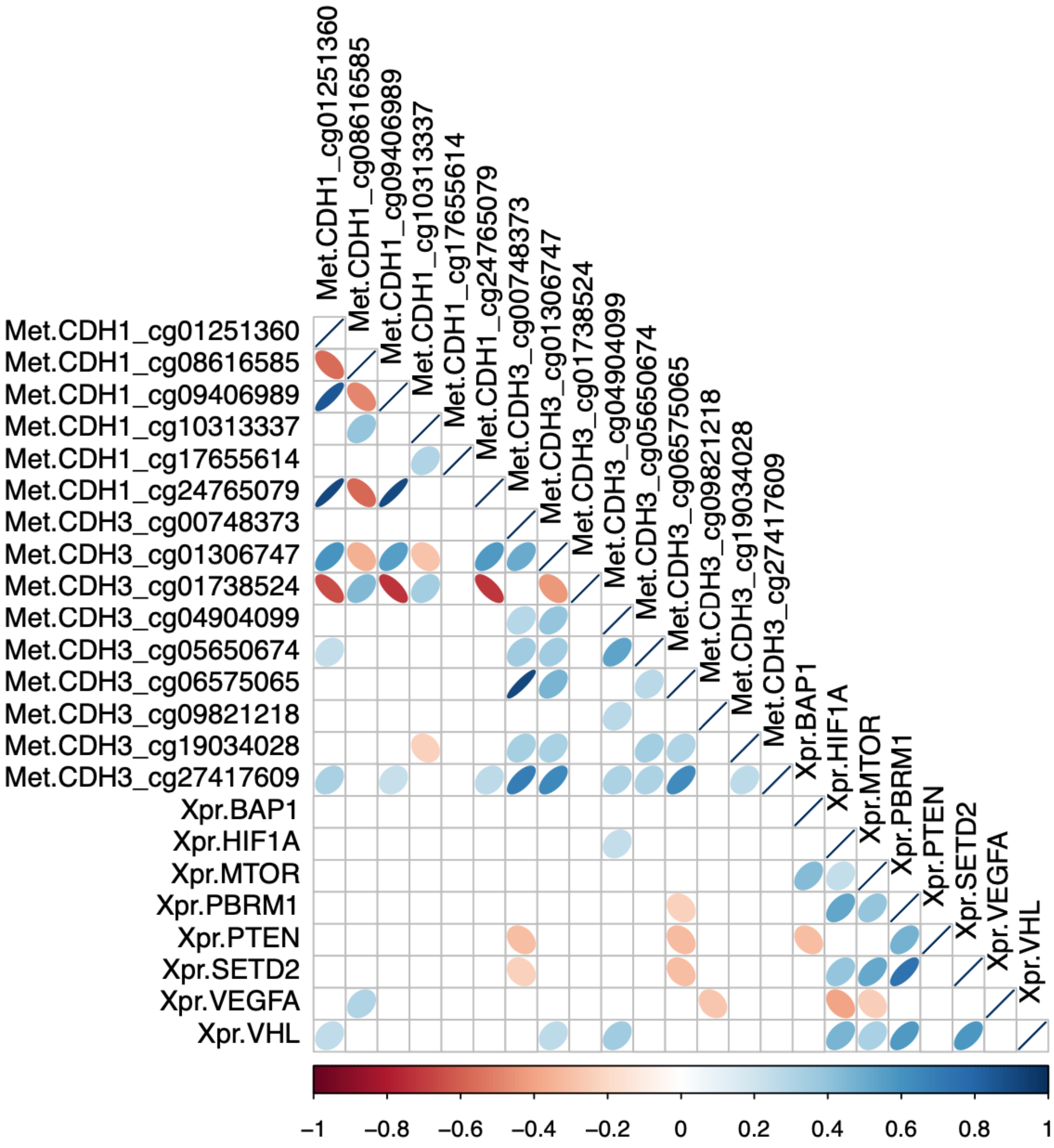

Figure S5. Presentation of Pearson's correlation analysis of associations between mRNA expression of the most frequently mutated genes (prefix "Xpr.") in ccRCC and CpG site-specific DNA methylation (prefix "Met.") of CDH3 according to the TCGA-KIRC study data (see Supplementary Figure S2 for explanation of graphic and presentation symbols). Note that expression of HIF1A and VHL as key RCC molecules largely do not show any association with methylation of CDH3 apart from a weak correlation with cg04904099 and cg01306747 ($R < 0.3$). In contrast, there seem to be weak negative correlations between SETD2 expression and CDH3 methylation (cg06575065 and cg00748373).

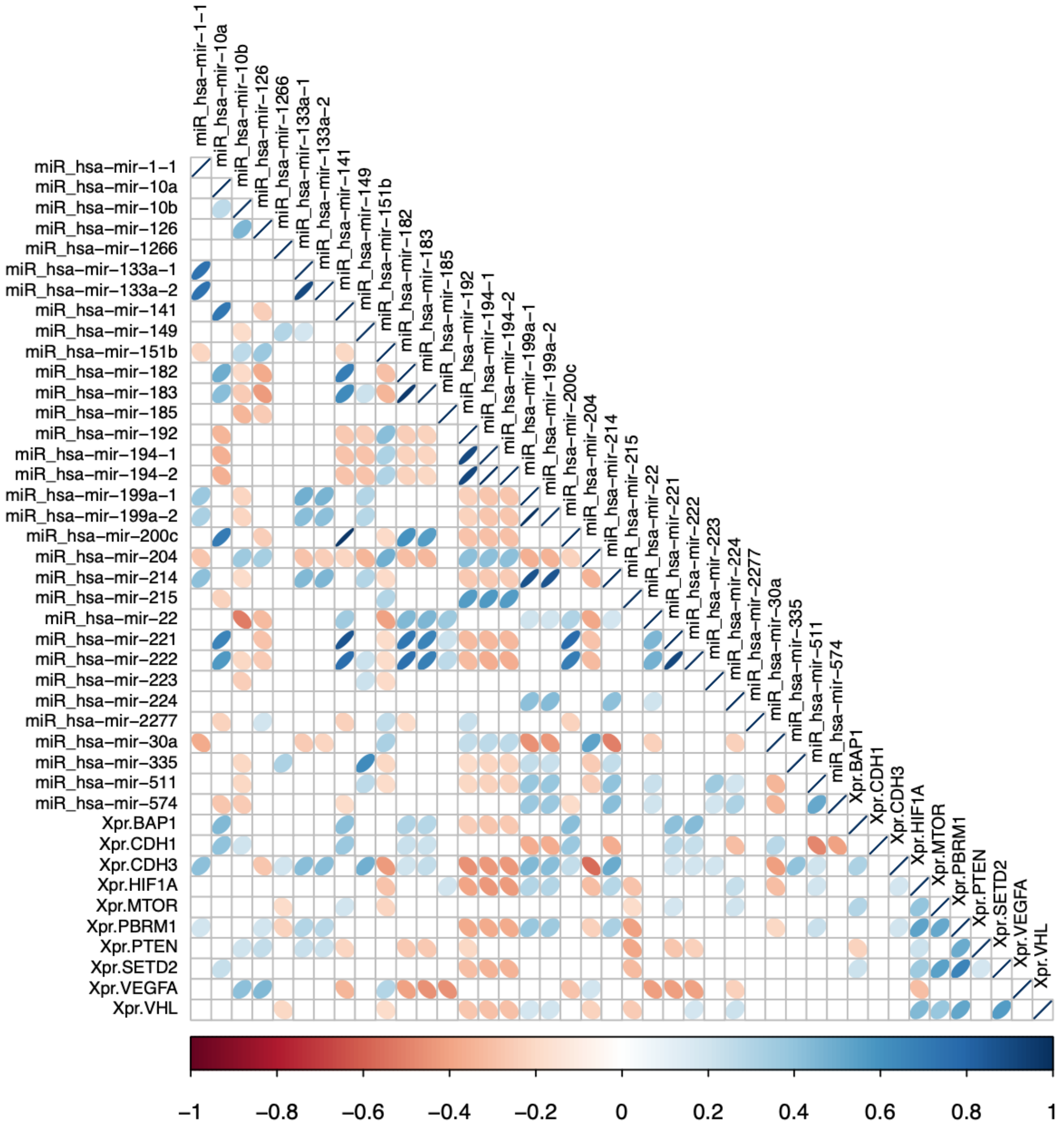

Figure S6. Presentation of Pearson's correlation analysis of associations between mRNA expression of the most frequently mutated genes (prefix "Xpr") in ccRCC and microRNA expression according to the TCGA-KIRC study data (see Supplementary Figure S2 for explanation of graphic and presentation symbols). Correlation analyses between mRNA expression of key RCC molecules as indicated and 536 microRNAs (miRs) based on TCGA-KIRC study data were carried out. Using a cut-off value of $R > 0.4$, the five miR correlations most strongly associated with the expression of key RCC molecules were used to create the correlation matrix. Of note, a substantial part of the mRNA expression of the key RCC molecules (BAP1, HIF1A, PBRM1, SETD2, VHL) as well as CDH3 all showed a pronounced or even strong association with miRs 204, 192, 194-1, and 194-2.

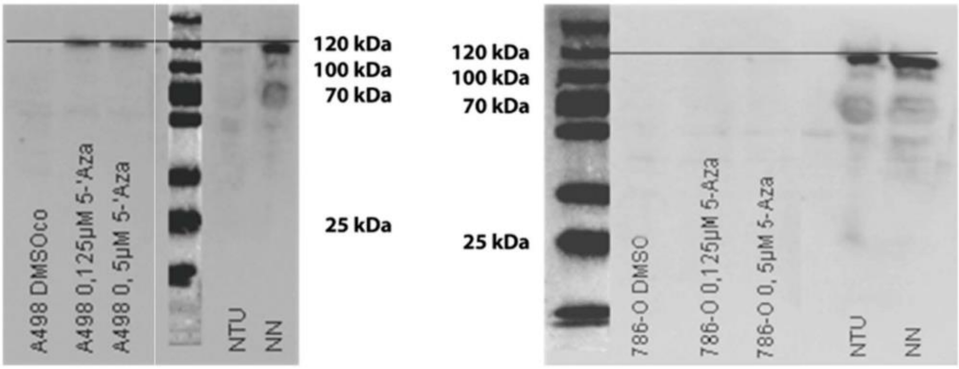

Figure S7. Western blotting analysis for detection of 5-AZA-induced re-expression of *CDH3* in RCC cell lines (NTU = tumor tissue; NN = tumor adjacent normal tissue). Note that visible immunopositivity was detected for A498 but not the 786-O cell line even though comparable total amounts of protein were analyzed (data not shown).

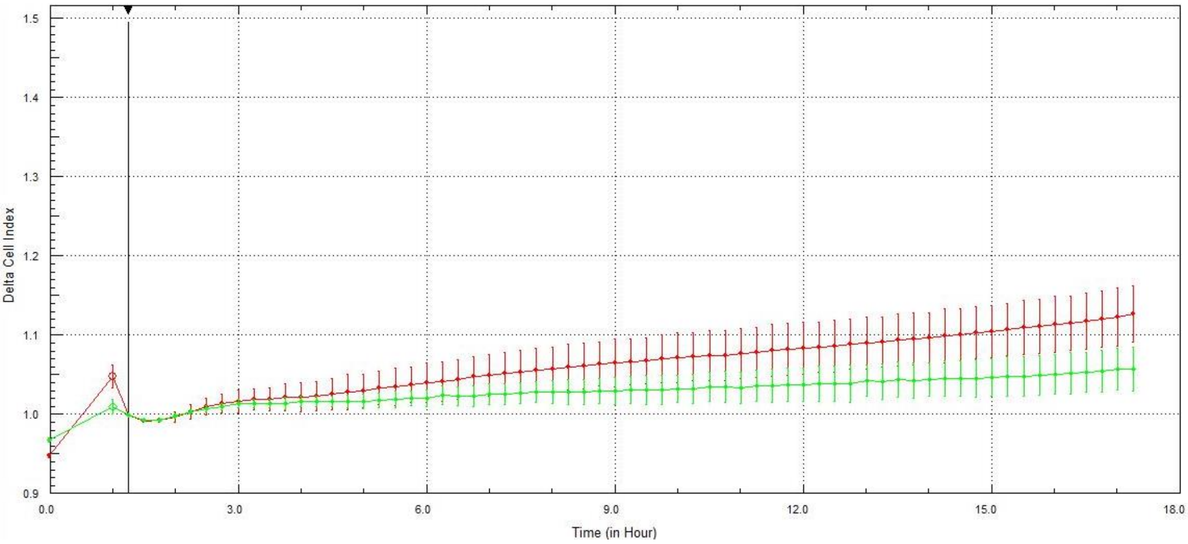

(**A**)

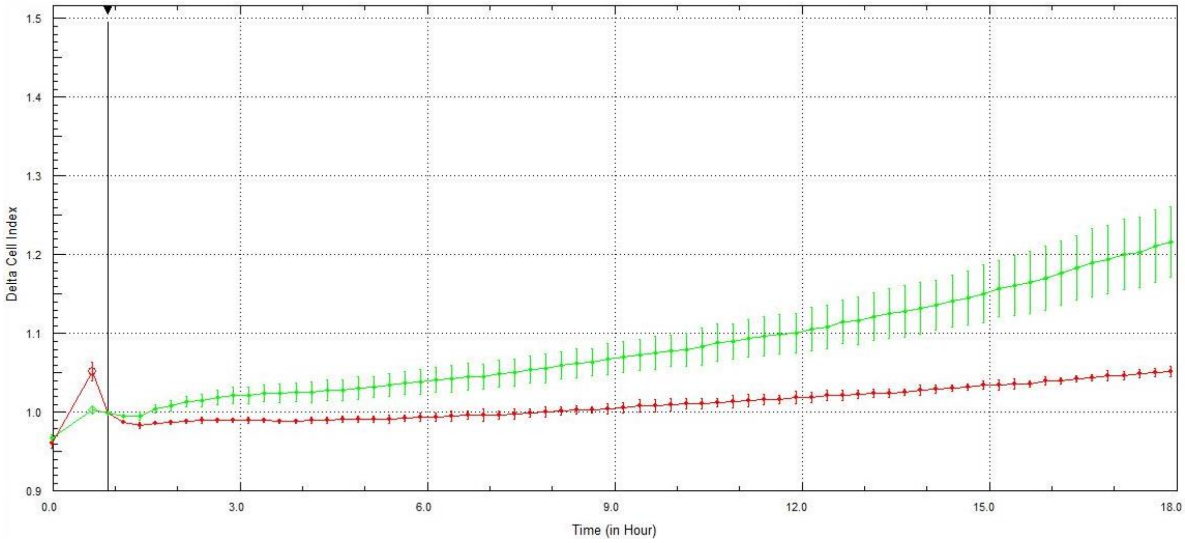

(**B**)

Figure S8. (**A**,**B**): Effects of siRNA-induced suppression of CDH1 (**A**) and CDH3 (**B**) expression on the Matrigel-transiting ability of cells as a surrogate for invasive potential of the bladder cancer cell line 5637 (see caption of Figure

7 for experimental details). Note that unlike the RCC experiments, the bladder cancer cell line tumor model showed substantial endogenous expression of CDH3. Suppression using on target plus siRNA mixture (green curve) against the Target plus control (red curve) exhibited a pronounced increase in invasive characteristics as detected by increased impedance following penetration of the Matrigel barrier.

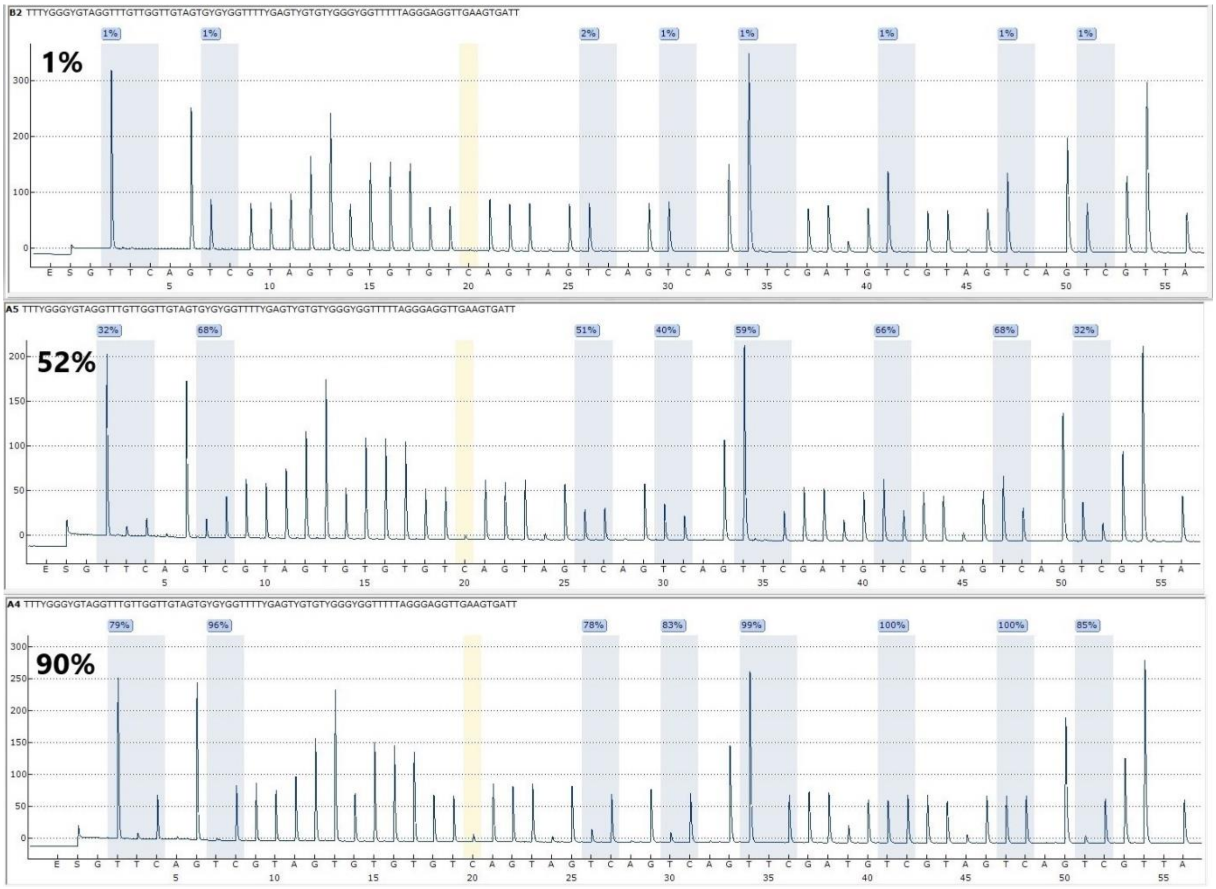

Figure S9. Exemplary demonstration of results obtained by pyrosequencing analysis of *CDH3* CGI methylation on chromosome 16. Blue shaded boxes show sequence variability following bisulfite conversion of DNA used for relative quantitation of T and C bases and determination of methylation percentage. Yellow shaded boxes indicate control C positions, providing a measure for individual sample conversion quality.
